# Multitrait Selection for Higher Agronomic and Tuber Yield–Related Traits in Tiger Nut (*Cyperus esculentus* L.) Genotypes

**DOI:** 10.1155/sci5/9458568

**Published:** 2025-06-23

**Authors:** Paul A. Asare, Adeyinka S. Adewumi, Patrick Twumasi, Emmanuel Afutu, Michael O. Adu

**Affiliations:** ^1^Department of Crop Science, University Post Office, University of Cape Coast, Cape Coast, Ghana; ^2^Soybean Breeding Unit, International Institute of Tropical Agriculture, PMB 5320, Ibadan, Nigeria

**Keywords:** *Cyperus esculentus*, factor analysis, genetic diversity, MGIDI index, multitrait selection

## Abstract

The tiger nut is a valuable crop that has been overlooked and underutilized. The tubers of this plant are highly valued for their nutritional benefits and health advantages. This study aimed to evaluate the genetic potential of 42 tiger nut genotypes using a multitrait index based on factor analysis and genotype-ideotype distance (MGIDI). The experiment involved a randomized complete block design with three replications, assessing 10 agronomic and tuber yield traits. The results showed significant variations among genotypes for all traits, which indicates the presence of genetic potential that can be harnessed for crop enhancement. The MGIDI index provided an increased total genetic gain of 78.58% for the desired traits. Specific genotypes, including PUT-b (FA1), ADU-b (FA2), OBR-B, ENK-B, ASU-b (FA3), and BEP-B (FA4), were identified as the top candidates for simultaneous improvement of the measured traits in tiger nut. This demonstrates the efficiency of the index in selecting superior genotypes, offering a data-driven approach to breeding programs aimed at optimizing tiger nut yield and quality. Furthermore, the identification of specific high-performing genotypes provides valuable genetic resources for future research and commercial cultivation. These findings contribute to the broader goal of improving food security and diversifying agricultural production. By promoting the genetic enhancement of tiger nut, this study supports its potential as a sustainable crop with significant nutritional and economic benefits.

## 1. Introduction

Tiger nut (*Cyperus esculentus* L.), also referred to as chufa or earth almond, is a perennial crop from the sedge family (Cyperaceae) [1]. With origins tracing back to ancient Egypt, this crop has been cultivated for millennia and has long been a vital source of nutrition and economic importance in various regions around the world [1–4]. Despite being underutilized, tiger nut has recently attracted more attention due to its exceptional nutritional value and versatile culinary uses. It is considered a superfood, rich in essential nutrients, dietary fiber, and healthy fats, which make it a promising crop for addressing global nutrition challenges, especially in low-resource areas [5–9]. Tiger nut tubers are an excellent source of minerals like iron, magnesium, and phosphorus, along with vitamins such as vitamin E and C [10, 11]. Moreover, its naturally gluten-free composition makes it a suitable option for people with gluten intolerance [12, 13].

Tiger nut cultivation holds significant promise, particularly in areas with harsh climatic conditions, as it is known for thriving in arid and semiarid regions with sandy soils [14, 15]. In light of growing concerns about food security and the need for sustainable agricultural practices, exploring ways to enhance the agronomic performance and tuber yield of this resilient crop is crucial. Although some studies have used morphological descriptors to assess the diversity within existing tiger nut germplasm [16–18], research on crop production and improvement remains limited. Farmers still rely on low-yielding landraces that are vulnerable to various stresses. The crop continues to face challenges in terms of productivity, quality, and adaptability to different environments.

To fully realize the potential of tiger nuts, new varieties with higher tuber yields and enhanced agronomic traits such as disease resistance, drought tolerance, and improved nutritional quality need to be developed. While previous research has explored morphological diversity in tiger nut germplasm [16–18], there is a lack of studies focusing on genetic enhancement through systematic breeding programs. Current cultivation relies on traditional landraces with low yields, limiting the crop's potential. Additionally, tiger nut is well adapted to harsh environments, but its yield stability and resilience to stress factors such as drought, pests, and diseases remain poorly understood. Research on identifying varieties with enhanced adaptability and nutritional quality is crucial for sustainable production. Targeted plant breeding programs that focus on selecting high-yielding genotypes with improved traits are essential. Multitrait selection, also known as selection index or multiple-trait selection, is an advanced breeding strategy that has been successful in enhancing crop performance [19]. Despite the success of multitrait selection methods in improving various crops [20–28], no prior studies have applied such techniques to tiger nut breeding. This gap hinders the identification of high-performing genotypes with superior agronomic traits, yield potential, and stress resilience.

Multitrait selection involves evaluating multiple desirable traits within a breeding population [19], enabling breeders to identify individuals with the optimal combination of characteristics [19]. While several linear selection indexes exist to help breeders identify superior genotypes [29], the multitrait genotype-ideotype distance index (MGIDI) focuses on selecting superior genotypes in breeding programs that evaluate multiple traits [19].

This present study effectively highlights the importance of tiger nut as a valuable but underutilized crop with exceptional nutritional and agronomic potential. It underscores the historical significance of tiger nut cultivation and its increasing recognition as a nutrient-rich food source. Its adaptability to arid conditions positions tiger nut as a promising candidate for sustainable agriculture, particularly in regions facing food insecurity and climate change challenges.

Furthermore, this study establishes a strong rationale for the study by identifying critical research gaps, particularly in the application of modern breeding techniques such as multitrait selection. By introducing the MGIDI index as an innovative tool for genotype selection, the study aims to address these gaps and provide a foundation for future breeding programs aimed at enhancing tiger nut yield, quality, and resilience. The study sets the stage for impactful research that could contribute to increasing the commercial viability, genetic improvement, and global adoption of tiger nut as a sustainable food crop.

This study sought to apply MGIDI to identify tiger nut varieties with superior agronomic and yield traits for future genetic improvement initiatives.

## 2. Materials and Methods

### 2.1. Experimental Site

The study took place at the Teaching and Research Farm of the School of Agriculture, University of Cape Coast, Ghana (5°07′7.6″N, 1°17′18.9″W; 15 m above sea level), situated in the Central Region, which encompasses both semideciduous forest and coastal savannah ecological zones. The area experiences a primary rainy season from April to June and a secondary rainy season from September to October, with dry harmattan conditions prevailing from November to February. The average temperature at the site is 25.8°C, and the average humidity is 86.3%. The field, previously used for cultivating eggplant, was ploughed and harrowed to a depth of around 30 cm. The soil at the site is classified as sandy loam, with a pH of 6.1, organic carbon content of 2.40%, available phosphorus at 776.8 μg/g, and potassium content measured at 0.072 cmol/kg.

### 2.2. Plant Materials and Experimental Design

**Figure 1:**
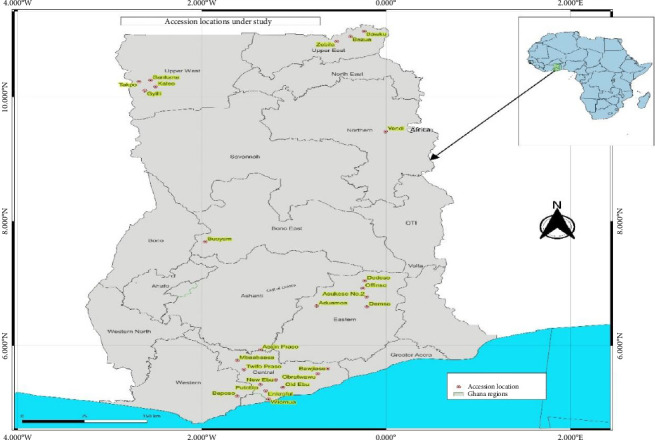
Map of Ghana showing regions and communities of collection of tiger nut genotypes.

This study utilized 42 tiger nut genotypes collected in 2021 from key growing regions. These included two genotypes from Bono East, 22 from the Central Region, seven from the Eastern and Upper East regions, three from the Northern Region, and one from the Upper West Region of Ghana (Supporting Table 1; Figure 1). Since the collected genotypes lacked passport data, they were named based on the first three letters of the town or community where they were collected, along with the tuber color. Genotypes from the same location with the same tuber color were further distinguished by numbering them. The tubers were placed in labeled, zip-locked polythene bags, transported to the Teaching and Research Farm at the University of Cape Coast, and then established in a field experiment.

The experiment followed a randomized complete block design (RCBD) with three replicates. The field layout randomization was generated using the “Agricolae” package in R software [30]. The total experimental area was 85 m^2^, measuring 5 m by 17 m, and was divided into 162 plots. Each plot measured 0.6 m by 1 m, and 10 tubers were planted per genotype with a spacing of 15 cm by 20 cm. Before planting, the genotypes were hydro-primed in disposable Styrofoam cups for 6 h to eliminate unviable tubers and promote sprouting. Plants were harvested 98 days after planting (DAP), though some genotypes showed signs of maturity at 85/86 DAP. Weeding and irrigation were conducted regularly as needed.

### 2.3. Data Collection

Agronomic and tuber yield traits were assessed, including percentage germination (PG), number of tillers per stand (NT), distance from the last tiller to the mother plant (DTP), plant height (PH), percentage inflorescence (PI), number of tubers per stand (NTS), number of rings per tuber (NRT), tuber length (LT), tuber width (WP), tuber shape (TS), and tuber size based on the weight of 100 tubers per genotype (WT). Data were collected from five randomly selected plants of each genotype per plot. The NT, DTP, PH, and PI were recorded at the time of flowering, while NTS was determined at harvest. Measurements for NRT, LT, WP, TS, and WT were taken 4 days post-harvest. TS was categorized into oval (< 1.3), ovoid (1.3–1.8), or oblong (> 1.8) based on the length-to-width (L/W) ratio [31]. Tuber weight was measured by weighing 100 randomly selected tubers per genotype using an electronic balance.

### 2.4. Statistical Analysis

The Lme4 package, an R package, was used to perform an analysis of variance (ANOVA) using a linear mixed model (LMM) [21]. The linear model used was as follows:

(1) $$\begin{array}{c} Y_{ij}=\mu +G_{i}+B_{j}+\varepsilon_{ij}, \end{array}$$

where *Y*_*ij*_ = value of the observed quantitative trait; *μ* = population mean; *G*_*i*_ = effect of the *i*th accession; *B*_*j*_ = effect of the *j*th complete block; and *ε*_*ij*_ = experimental error. Accessions were considered fixed effects, and blocks were considered random. Pearson's correlation coefficients for quantitative traits were calculated using the Corrplot R package Version 0.84 [32]. Principal component analysis (PCA) was employed to evaluate the contribution of these traits, utilizing the FactoMineR and Factoextra packages in R [30]. The relationships among the 42 tiger nut genotypes were assessed with the cluster package, and hierarchical clustering was visualized using the ape package. The silhouette method, implemented in FactoMineR and Factoextra, was used to determine the optimal number of clusters [30]. Variance components for each quantitative trait were estimated from the expected mean square (EMS) in the ANOVA, using lmerTest and lme4 in the R package [30]. Broad-sense heritability, along with genotypic coefficients of variation (GCV) and phenotypic coefficients of variation (PCV), was calculated based on these variance components.

(2) $$\begin{array}{c} H^{2}=\left(\frac{\delta_{g}^{2}}{{\delta_{g}^{2}+\delta }_{p/n\text{⁣}}^{2\text{⁣}}}\right)\times 100,\\ \text{Phenotypic coefficient of variance}\left(\text{PCV}\right)=\frac{\sqrt{\delta_{p\text{⁣}}^{2}}}{\text{Grand mean}}\times 100,\\ \text{Genotypic coefficient of variance}\left(\text{GCV}\right)=\frac{\sqrt{\delta_{g}^{2}}}{\text{Grand mean}}\times 100, \end{array}$$

where *δ*_*g*⁣_^2^ is the genotypic variance and *δ*_*p*⁣_^2^ is the phenotypic variance. Shabanimofrad et al. [33] categorized the estimated values of PCV and GCV as 0%–10% for low, 10%–20% for intermediate, and greater than or equal to 20% for high. Broad-sense heritability (*h*^2^) was categorized as 0%–29% for low, 30%–60% for intermediate, and greater than 60% for high.

The MGIDI is based on four main steps: (i) rescaling all traits to a range of 0–100, (ii) applying factor analysis to account for correlations and reduce data dimensionality, (iii) designing an ideotype using known or desired trait values, and (iv) calculating the distance between each genotype and the ideotype. To rescale the traits, equation (3) was applied.

(3) $$\begin{array}{c} {rX}_{ij}=\frac{\eta_{nj}}{\eta_{oj}}-\frac{\varphi_{nj}}{\varphi_{oj}}*\left({ϴ}_{ij}-\eta_{nj}\right)+\eta_{nj}, \end{array}$$

where *η*_*nj*_ and *φ*_*nj*_ are the new maximum and minimum values for the trait *j* after rescaling, respectively; *φ*_*oj*_ and *φ*_*oj*_ are the original maximum and minimum values for the trait *j*, respectively; and *ϴ*_*ij*_ is the original value for the *j*th trait of the *i*th genotype/treatment.

The values for *η*_*nj*_ and *φ*_*nj*_ are chosen as follows: For the traits in which negative gains are desired, *η*_*nj*_ = 0 and *φ*_*nj*_ = 100 should be used. For the traits in which positive gains are desired, *η*_*nj*_ = 100 and *φ*_*nj*_ = 0 [34, 35]. In the rescaled two-way table (*rX*_*ij*_), each column has a 0–100 range that considers the desired sense of selection (increase or decrease) and maintains the correlation structure of the original set of variables.

The factor analysis and ideotype design (MGIDI) index were used to rank the genotypes based on multiple traits, eliminating the issue of multicollinearity [36]. A radar chart was created using the radar chart function from the fmsb package [28]. The predicted genetic gain, SG (%), for each trait was calculated based on the MGIDI index, considering a selection intensity of *α*%, as follows:

(4) $$\begin{array}{c} \text{SG}\left(\%\right)=\frac{\left({Ⴟ}_{s}-{Ⴟ}_{o}\right)h^{2}}{{Ⴟ}_{o}}, \end{array}$$

where *Ⴟ*_*s*_ is the mean of the selected genotypes, *Ⴟ*_*o*_ is the mean of the original population, and *h*^2^ is the heritability.

## 3. Results

### 3.1. Variations in Agronomic and Tuber-Related Traits Among the Evaluated Germplasm

The ANOVA showed a significant (*p* < 0.001 or *p* < 0.05) genotype effect for all the evaluated traits (Table 1). The overall mean, minimum, and maximum values and coefficients of variation of evaluated agronomic and tuber yield traits are presented in Table 1. The hundred tuber weight varied from 31.02 to 201.65 g, averaging 133.46 g (Table 1; Supporting Table 2). The average NTS was 39 tubers, ranging from ∼13 to ∼ 76 tubers. The LT ranged from 8.82 to 30.81 mm with a mean value of 19.46 mm, while the WP varied from 7.96 to 17.22 mm with an average value of 14.22 mm (Table 1; Supporting Table 2). The coefficients of variation ranged from 0.19% for hundred tuber weight to 44.91% for the NTS (Table 1; Supporting Table 2).

### 3.2. Variance Components and Broad-Sense Heritability of Agronomic and Tuber Yield Traits

High GCV (≥ 20%) were observed for traits such as hundred tuber weight, length of tuber, number of tillers, PG, and TS. In comparison, moderate GCV (11%–19%) were observed for traits DTP, NTS, PH, and WT, and low GCV ≤ 10% was observed for the NRT (Table 2). High PCV (≥ 20%) were recorded for most of the traits evaluated except for WP with moderate PCV (11%–19%) and NRT with low PCV (≤ 10%) (Table 2). All the evaluated traits recorded high broad-sense heritability (≥ 60%) and ranged from 81% for the NTS to 98% for hundred tuber weight (Table 2).

### 3.3. Contribution of Evaluated Agronomic and Tuber Yield Traits to Principal Components (PCs)

**Figure 2:**
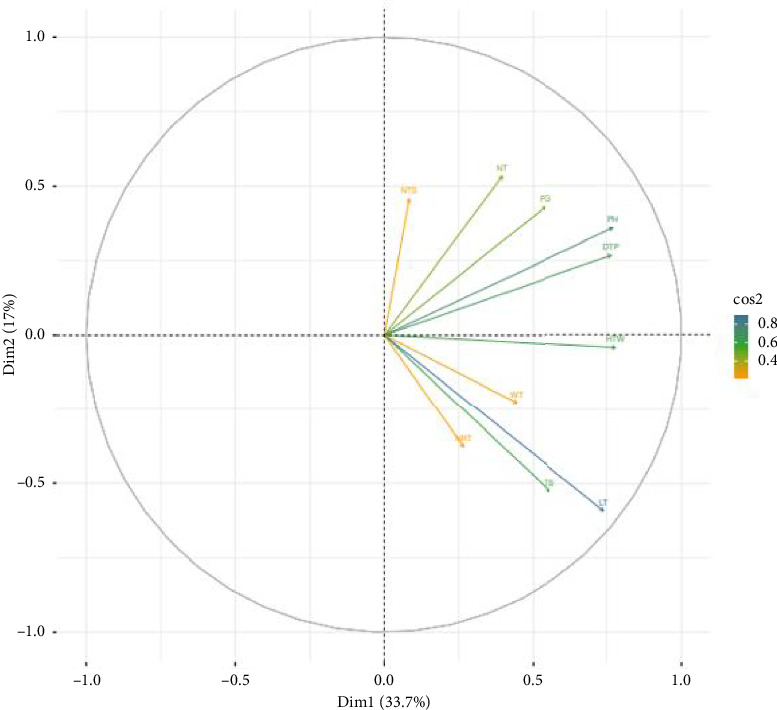
Plot showing the total contribution of variables accounting for the variability in Dim1 and Dim2; acronyms for traits are defined in Table 1.

The first four PCs with eigenvalues of more than 1.00 accounted for ∼78% of the total variation (Table 3). Principal component one (PC1) explained 33.70% of the total variation. It showed a highly significant association with traits such as distance of the last tiller from the main plant (*r* = −0.765), PH (*r* = −0.769), LT (*r* = −0.68), and hundred tuber weight (*r* = −0.779) (Table 3 and Figure 2). The second principal component (PC2) contributed 17% of the total variation and revealed a significant positive correlation with the NT (*r* = 0.534) (Table 3 and Figure 2). Approximately 15% of the total variation was detected in the third principal component (PC3) and revealed a significant positive contribution to NRT (*r* = 0.533) and WP (*r* = 0.657) but had negative relationship with the TS (*r* = −0.585) (Table 3 and Figure 2). PC 4 explained a total variation of 12.80% with a significant positive contribution from PG (*r* = 0.611) but a significant negative relationship from the NTS (*r* = −0.509) (Table 3 and Figure 2).

### 3.4. Relationships Among the Evaluated Agronomic and Tuber Yield Traits

**Figure 3:**
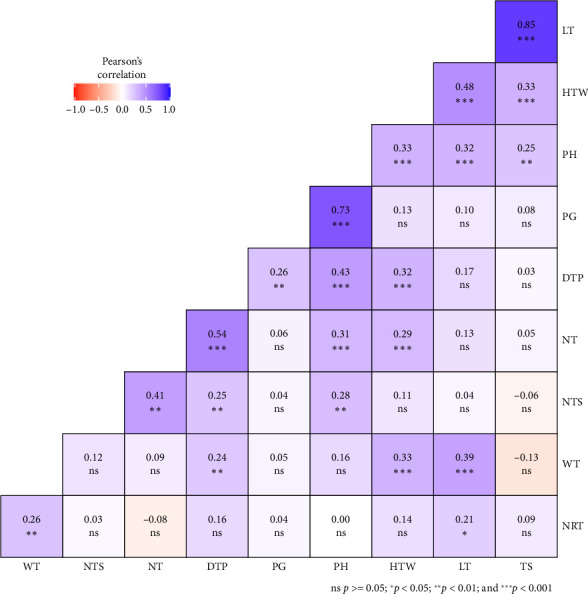
Relationship among the evaluated agronomic and tuber yield traits. Acronyms for traits are defined in Table 1; ^∗∗∗^ = *p* < 0.001; ^∗∗^ = *p* < 0.01; ^∗^ = *p* < 0.05; ns = *p* > 0.05.

Significant correlations were observed among the 10 agronomic and tuber yield traits evaluated (Figure 3). Hundred tuber weight had significant and positive correlations with TS, LT, WP, stem diameter, NT, distance of the last tiller from the main plant, and PH (*p* < 0.001). PG was positively correlated to the distance of the last tiller from the main plant and PH (*p* < 0.001). The NTS was significantly and positively correlated with PH (*p* < 0.01), distance of the last tiller from the main plant (*p* < 0.01), and NT (*p* < 0.001). NT revealed significant and positive correlations with PH (*p* < 0.001) and distance of the last tiller from the main plant (*p* < 0.001). PH was significantly and positively correlated with the distance of the last tiller from the main plant (*p* < 0.001).

### 3.5. Path Analysis Among the Evaluated Agronomic and Tuber Yield Traits

**Figure 4:**
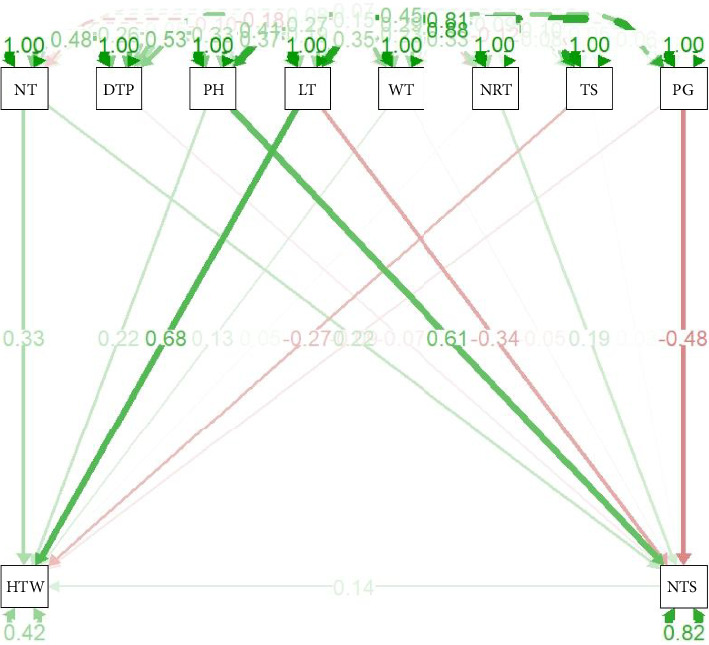
Path analysis evaluated 10 agronomic and tuber yield traits using hundred tuber weight and number of tubers per stand as dependent variables. Acronyms for traits are defined in Table 1.

Direct path analysis revealed positive contributions of NT (*r* = 0.33), PH (*r* = 0.22), LT (*r* = 0.68), WP (*r* = 0.13), and NTS (*r* = 0.14) to hundred tuber weight, while TS (*r* = −0.27) had negative contribution to hundred tuber weight (Figure 4). Path coefficient analysis also showed positive contributions of NT (*r* = 0.22), PH (*r* = 0.61), and NRT (*r* = 0.19) to NTS, while LT (*r* = −0.34), WP (*r* = −0.05), and PG (*r* = −0.48) had negative contributions to NTS (Figure 4).

### 3.6. Cluster Membership of 42 Tiger Nut Genotypes

**Figure 5:**
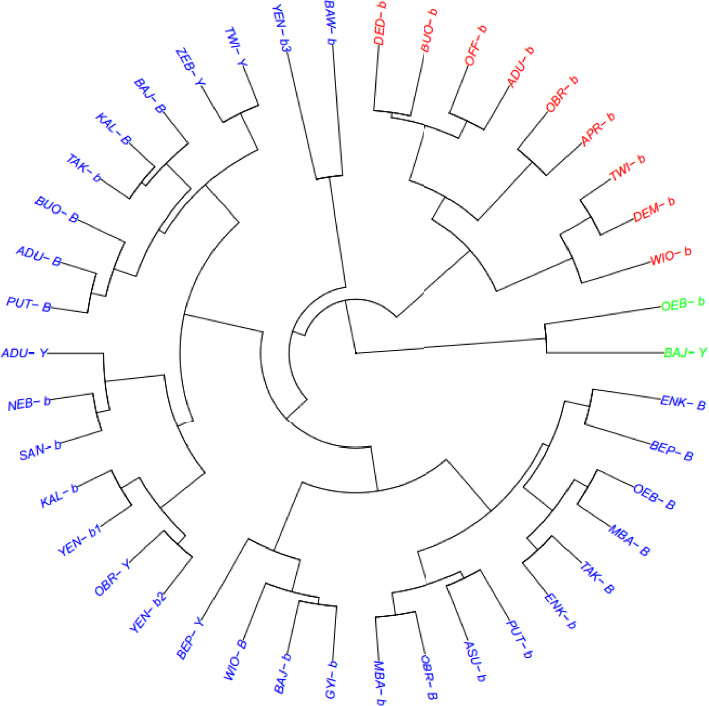
Hierarchical dendrogram showing grouping patterns of tiger nut genotypes using 10 agronomic and tuber yield traits based on Gower dissimilarity coefficients. Cluster 1—red, Cluster 2—blue, and Cluster 3—green.

Based on the Gower dissimilarity matrix, the tiger nut genotypes were grouped into three clusters: cluster 1 containing nine genotypes, cluster 2 containing 31 genotypes, and cluster 3 containing two genotypes (Table 4; Figure 5). Cluster 1 comprised genotypes that showed significant performance for hundred tuber weight, length, and PH, distance of the last tiller from the main plant, PG, and TS (Table 4). Cluster 2 comprises genotypes with moderate performance for hundred tuber weight, length, and shape. This cluster also showed significant performance for PH, distance of the last tiller from the main plant, and PG (Table 4). The three clusters showed equal performance for the NRT, NT, NTS, and WP (Table 4).

### 3.7. Factor Analysis and Predicted Selection Gain

The tiger nut ideotypes were determined by estimating the genetic correlation of each factor using the MGIDI index. The MGIDI index identified four factors. Factor 1 is linked to the NT and tubers per stand—factor 2 correlates with hundred tuber weight, length, and shape. Factor 3 is associated with the NRT and WP, while factor 4 is related to the percentage of germination, distance of the last tiller from the main plant, and PH (Table 5). The average communality and uniqueness accounted for 78% and 22% of the total genetic variability in the dataset, respectively (Table 5). All the evaluated 10 traits had desired genetic gains using the MGIDI index (Table 5). The predicted genetic gain ranged from 0.42% for PG to 22.80% for hundred tuber weight, with total expected MGIDI genetic gains of 78.58% for the assessed multitrait desired for increase (Table 5).

**Figure 6:**
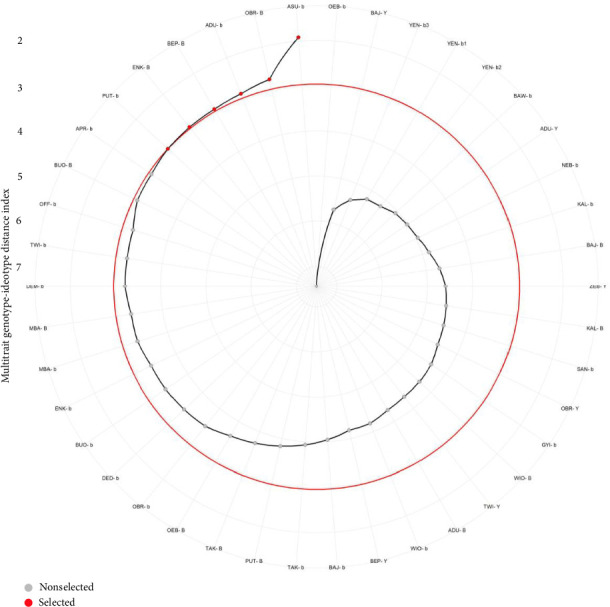
Tiger nut genotype ranking showing selected accessions using the multitrait genotype-ideotype index (MGIDI). The selected accessions are red dots, while the unselected in accessions are black dots. The red circle represents the cut point according to the selection pressure.

**Figure 7:**
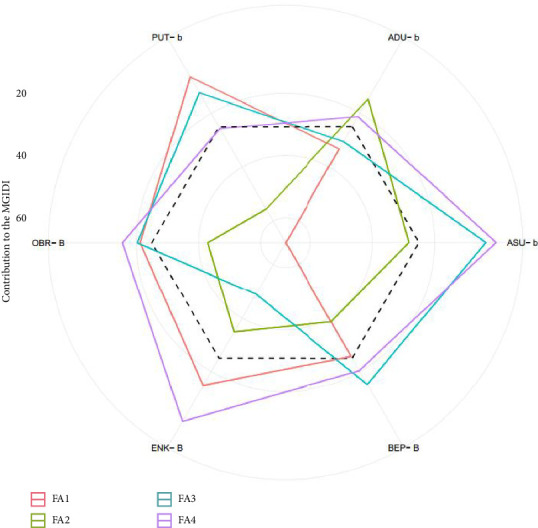
The strength and weakness view of the selected genotypes is shown as the proportion of each factor on the computed multitrait genotype-ideotype index (MGIDI). The smaller the proportion explained by a factor (positioned nearer to the outer edge), the more closely the traits within that factor align with the ideotype. The black broken circle at the center shows the theoretical value if all the factors were to contribute equally.

Out of the 42 tiger nut genotypes that were evaluated, the MGIDI index identified six genotypes (ASU-b, OBR-B, ADU-b, BEP-B, ENK-B, and PUT-b) as high-performing genotypes for multiple traits (Figure 6). When the selected genotypes were evaluated for their strengths and weaknesses, it was revealed that FA1 made the most significant contribution to genotype PUT-b, FA2 had the highest contribution to genotype ADU-b, FA3 had the highest contribution to genotypes OBR-B, ENK-B, and ASU-b, and FA4 had the highest contribution to genotype BEP-B (Table 6; Figure 7).

## 4. Discussion

This study evaluated the genetic potential of 42 tiger nut (*Cyperus esculentus*) genotypes across multiple traits, revealing significant variations in agronomic and tuber yield traits. The high genotype effect observed on all evaluated traits evaluated in this study indicates strong genetic influence on agronomic and tuber yield characteristics. This finding aligns with previous studies that emphasize the role of genotype in determining yield-related traits in tuber crops [17, 18]. A high CV for tuber number suggests greater environmental and genetic influence on this trait, making it an important selection criterion for breeding [22, 26]. In contrast, the low CV for hundred tuber weight indicates more stability, implying that this trait is relatively less affected by environmental fluctuations [22, 26]. The wide variation observed suggests opportunities for genetic improvement through breeding programs aimed at enhancing yield, tuber size, and overall productivity [22, 26].

Heritability is a key factor in crop improvement, influencing the predictability and efficiency of trait selection, environmental stability, and the selection of complex traits while minimizing the effects of random factors [37]. The presence of high GCV in key yield-related traits, such as hundred tuber weight and LT, suggests that genetic improvement through selection could lead to significant yield gains [20, 26]. These findings align with previous research indicating that high GCV values reflect substantial genetic variability, which is essential for effective selection in breeding programs [20, 26]. The high PCV (≥ 20%) recorded for most traits indicate that, in addition to genetic factors, environmental conditions significantly influence trait expression [20, 26]. The high broad-sense heritability found across all traits in this study indicates a strong genetic influence, making these traits suitable for future breeding efforts. High heritability values suggest that selection based on phenotypic performance will be effective in improving these traits [20, 26]. The exceptionally high heritability of hundred tuber weight implies that this trait is primarily governed by genetic factors, making it a reliable selection criterion in breeding programs [20, 26]. Similarly, the high heritability of the NTS suggests that genetic selection can significantly enhance yield potential [20, 26].

In plant breeding, multivariate analysis tools, such as PCA and clustering, offer systematic, data-driven methods for improving crop varieties. The PCA results indicate that the first four PCs, with eigenvalues greater than 1.00, accounted for approximately 78% of the total variation, highlighting the effectiveness of PCA in reducing the dimensionality of the dataset while retaining most of the variation [21, 38, 39]. This level of explained variance aligns with previous studies on genetic diversity in tuber crops, where PCA effectively grouped traits with high genetic contributions [21, 38, 39]. PCA results provide valuable insights into trait relationships in tuber crops. The strong associations between specific traits highlight potential selection criteria for breeding programs aimed at optimizing yield, tuber morphology, and plant architecture. The strong negative associations observed with the distance of the last tiller from the main plant, PH, LT, and hundred tuber weight suggest that genotypes with taller plants and longer tubers tend to have lower hundred tuber weight, potentially due to resource allocation trade-offs. The positive correlation exhibited by NT suggests that PC2 primarily captures variation associated with tillering capacity, which is a key trait in yield determination as it influences the number of tubers produced per plant. A higher number of tillers are often associated with increased tuber production, but this relationship can vary based on genotype and environmental factors. The positive association between WP and the NRT indicates that genotypes producing wider tubers tend to have more internal rings, which may influence processing and storage qualities.

These correlation results highlight the intricate relationships among agronomic and yield traits in tuber crops. The strong associations between hundred tuber weight, PH, tiller number, and tuber dimensions suggest that selecting for robust plant growth can enhance overall yield potential [20]. Furthermore, the positive correlations between germination percentage, PH, and tiller spread emphasize the importance of early plant vigor in determining final yield outcomes [20].

The path analysis results indicate that certain traits have strong positive contributions, while others exhibit negative influences, reflecting complex genetic and physiological interactions [20]. The path analysis results emphasize the importance of key morphological traits in determining tuber yield and quality [20]. Traits such as PH, NT, and LT have strong positive influences on hundred tuber weight and the NTS, making them valuable selection criteria in breeding programs. However, the negative contributions of certain traits, such as TS and PG, highlight the need for a balanced selection strategy to optimize both yield and tuber quality.

The clustering analysis based on the Gower dissimilarity matrix successfully categorized tiger nut genotypes into three distinct groups, each with unique agronomic characteristics. Cluster 1 genotypes demonstrated superior performance in key yield-related traits, making them prime candidates for selection in breeding programs. Cluster 2 contained the majority of genotypes, indicating a broad genetic base that may be useful for maintaining diversity, while Cluster 3 represented rare genotypes with potentially valuable traits. These findings highlight the importance of clustering analysis in identifying promising genotypes for crop improvement and ensuring genetic diversity in breeding populations.

The use of the MGIDI provided an effective approach for selecting superior tiger nut ideotypes by estimating the genetic correlation of key agronomic traits [19, 40]. The identification of four distinct genetic factors highlights the complexity of trait interactions and their contributions to overall yield and plant performance (Table 5). The MGIDI index serves as a valuable selection tool by integrating multiple traits into a single selection criterion, optimizing breeding efficiency [19, 40]. Factor 1, which is associated with the NT and tubers per stand, emphasizes the importance of vegetative vigor in determining yield potential (Table 5). A higher number of tillers have been linked to increased photosynthetic capacity and resource allocation, ultimately leading to greater tuber production. Similarly, the NTS directly influences overall yield, making this factor critical for breeding high-yielding tiger nut varieties. Factor 2, which correlates with hundred tuber weight, LT, and TS, underscores the role of tuber size and morphology in determining marketable yield (Table 5). The significant contribution of hundred tuber weight (22.80% genetic gain) suggests that selecting for heavier and well-shaped tubers can substantially enhance yield and quality [20, 22, 23, 26]. Studies on tuber crops such as potato have also shown that larger and more uniform tubers fetch higher market value, making these traits important selection criteria in commercial breeding programs [23, 24]. Factor 3, which is linked to the NRT and WP, provides insights into tuber structural characteristics (Table 5). While these traits may not directly impact overall yield, they contribute to tuber quality and consumer preference [20]. The genetic gains observed for these traits suggest that they can be improved through targeted breeding, ensuring better uniformity in tuber dimensions [26].

Factor 4, associated with PG, distance of the last tiller from the main plant, and PH, highlights the importance of early establishment and plant vigor in achieving high yields (Table 5). The positive genetic gain for PG (0.42%) indicates that improvements in seed viability and early growth potential can enhance overall plant performance [38].

The total genetic variability explained by the MGIDI index was 78%, with 22% accounted for by uniqueness (Table 5). This suggests that the selected traits capture the majority of genetic variation in the dataset, ensuring that the MGIDI index effectively prioritizes important agronomic traits [26, 37–39, 41]. The total expected genetic gains of 78.58% across the assessed multitrait indicate strong selection efficiency, which can significantly improve breeding outcomes in tiger nut improvement programs [26, 38, 39].

The MGIDI index effectively identified six high-performing tiger nut genotypes: ASU-b, OBR-B, ADU-b, BEP-B, ENK-B, and PUT-b, demonstrating its robustness in selecting superior genotypes for multiple agronomic and yield-related traits (Figure 6). The identification of these genotypes suggests that they exhibit desirable genetic characteristics and could serve as potential candidates for further breeding and commercialization [20, 26, 38, 39].

The identification of these six high-performing genotypes highlights the effectiveness of the MGIDI index in capturing multitrait genetic potential (Table 6; Figure 7). The varying contributions of the four genetic factors suggest that each genotype has unique strengths, which can be strategically utilized in breeding programs. For instance, genotypes excelling in FA1 (PUT-b) can be used for improving plant vigor and tuber multiplication, while those with high FA2 contributions (ADU-b) can be targeted for improving tuber weight and shape [19, 40]. Moreover, the presence of multiple genotypes with strong FA3 and FA4 contributions suggests that breeding strategies can focus on balancing tuber structural traits with plant growth characteristics to develop high-yielding and market-preferred varieties [19, 40]. The selection of genotypes with high PG and PH (BEP-B) also underscores the importance of early plant establishment in achieving high productivity [19, 40].

## 5. Conclusion

Our study has shed light on the untapped potential of tiger nut (*Cyperus esculentus* L.) genotypes for simultaneously improving agronomic and tuber yield–related traits. Using the innovative MGIDI, we have demonstrated the effectiveness of multitrait selection in harnessing the genetic potential of this crop. Significant genotype variations were observed across 10 key agronomic and tuber yield traits, indicating high genetic diversity within the tiger nut germplasm, demonstrating the importance of genetic factors in trait expression. High broad-sense heritability (≥ 60%) was recorded across all traits, suggesting that these traits are primarily governed by genetic factors and can be effectively improved through breeding programs. Notably, hundred tuber weight exhibited the highest heritability (98%), making it a reliable selection criterion for yield improvement. PCA revealed that the first four PCs explained approximately 78% of the total variation, with PC1 alone accounting for 33.70%. Traits such as PH, LT, hundred tuber weight, and distance of the last tiller from the main plant significantly contributed to PC1. These findings suggest that these traits are major determinants of phenotypic diversity and should be prioritized in selection programs. Correlation and path coefficient analyses identified critical relationships between yield-related traits. Hundred tuber weight was positively correlated with key morphological traits such as TS, LT, WP, and PH (*p* < 0.001), indicating their collective contribution to yield. Direct path analysis further confirmed that traits like LT, PH, and NT positively influenced hundred tuber weight, while TS negatively affected it. Such insights provide a robust foundation for designing effective breeding strategies aimed at improving yield and quality. Clustering analysis based on the Gower dissimilarity matrix classified the genotypes into three distinct clusters, with Cluster 1 comprising genotypes that exhibited superior performance for hundred tuber weight, LT, PH, PG, and TS. This classification provides useful information for selecting and crossbreeding genotypes with complementary traits to enhance overall productivity. We recorded a substantial increase in total genetic gain achieved by applying the MGIDI index, leading to a gain of 78.58% for the desired traits. Specific genotypes, such as PUT-b (FA1), ADU-b (FA2), OBR-B, ENK-B, ASU-b (FA3), and BEP-B (FA4), emerged as promising candidates for comprehensive trait improvement in tiger nut. These genotypes exhibited high performance across the measured traits, making them prime candidates for further breeding efforts and developing superior tiger nut varieties. Our findings emphasize the significance of tiger nut as a crop with untapped potential and underscore the value of adopting multitrait selection strategies, particularly the MGIDI index, in enhancing this crop's performance. Simultaneously improving multiple agronomic and yield-related traits is vital in meeting the growing demand for nutritious and sustainable food sources. As tiger nut gains recognition for its nutritional and health benefits, versatility in culinary applications, and adaptability to diverse environments, our study contributes to the ongoing efforts to harness the full genetic potential of this crop.

## Figures and Tables

**Table 1:** Mean squares, means, and coefficients of variation for agronomic and tuber yield traits evaluated among tiger nut genotypes.

Traits	Block	Genotypes	Residual	Mean	Minimum	Maximum	CV (%)
DTP (cm)	49.72^∗∗∗^	9.528^∗∗^	4.91	9.45	3.4	12.6	23.44
HTW (g)	1.64^∗∗∗^	4554.42^∗∗∗^	0.06	133.46	31.02	201.65	0.19
LT (mm)	271.82^∗∗∗^	76.12^∗∗∗^	9.28	19.46	8.82	30.81	15.65
NRT (count)	0.10^ns^	0.62^∗∗∗^	0.07	5.75	5.00	6.67	4.62
NT (count)	274.65^∗∗∗^	53.72^∗∗∗^	16.88	13.09	2.6	24.2	31.38
NTS (count)	3230.89^∗∗∗^	504.74^∗^	306.83	39	12.93	75.67	44.91
PG (%)	1736.89^∗^	1323.49^∗∗∗^	465.13	70.04	12.22	100	30.79
PH (cm)	1569.56^∗∗^	806.06^∗∗∗^	303.21	91.77	27.8	113.33	18.97
TS	0.21^∗^	0.36^∗∗∗^	0.06	1.37	0.98	2.45	18
WT (mm)	60.94^∗∗∗^	11.33^∗∗∗^	1.45	14.22	7.96	17.22	8.48

*Note:* NT = number of tillers per plant; DTP = distance of the last tiller from the main plant.

Abbreviations: CV = coefficients of variation, HTW = hundred tuber weight, LT = length of tuber, NRT = number of rings per tuber, ns = not significant, NTS = number of tubers per stand, PG = percentage germination, PH = plant height, PI = percentage inflorescence, TS = tuber shape, and WT = width of tuber.

^∗^ = *p* < 0.05.

^∗∗^ = *p* < 0.01.

^∗∗∗^ = *p* < 0.001.

**Table 2:** Variances and broad-sense heritability of agronomic and tuber yield traits.

Traits	*δ* ^2^ *g*	*δ* ^2^ *p*	GCV	PCV	*H* ^2^
DTP (cm)	1.18	7.16	0.11	0.28	0.87
HTW (g)	1520	1520.1	0.29	0.29	0.98
LT (mm)	20.19	35.72	0.23	0.31	0.96
NRT (count)	0.18	0.25	0.07	0.09	0.97
NT (count)	10.23	33.25	0.24	0.44	0.93
NTS (count)	42.76	419.21	0.17	0.52	0.81
PG (%)	276	771.4	0.24	0.40	0.94
PH (cm)	157.6	491	0.14	0.24	0.93
TS	0.1	0.17	0.23	0.30	0.96
WT (mm)	2.82	5.69	0.12	0.17	0.95

*Note:* Acronyms for traits are defined in Table 1; *δ*^2^*g* = genotypic variance; *δ*^2^*p* = phenotypic variance; *H*^2^: broad-sense heritability.

Abbreviations: GCV = genotypic coefficient of variation; PCV = phenotypic coefficient of variation.

**Table 3:** Principal component analysis of agronomic and tuber yield traits evaluated among tiger nut genotypes.

Traits	PC1	PC2	PC3	PC4
PG (%)	−0.5433	0.4317	0.2608	**0.6108**
NT (count)	−0.3971	**0.5341**	−0.3903	−0.3905
DTP (cm)	**−0.765**	0.2674	0.2376	−0.149
PH (cm)	**−0.7699**	0.3592	0.02507	0.4216
NTS (count)	−0.08372	0.4568	−0.1946	**−0.5094**
NRT (count)	−0.2678	−0.3808	**0.5325**	−0.02546
LT (mm)	**−0.7392**	−0.05909	−0.2599	0.02583
WT (mm)	−0.4453	−0.2287	**0.6569**	−0.2731
TS	−0.5567	−0.5241	**−0.5852**	0.1781
HTW (g)	**−0.7796**	−0.0431	−0.1138	−0.3504
Eigenvalues	3.37	1.70	1.45	1.28
Variance (%)	33.70	17.00	14.50	12.80
Cum. variance (%)	33.70	50.00	65.30	78.10

*Note:* Acronyms for traits are defined in Table 1. PC1–PC4: principal components. The bold values indicate the traits that has the most significant contributions at each PC.

**Table 4:** Cluster description for 10 agronomic and tuber yield traits of tiger nut genotypes.

Traits	Cluster 1 (9)	Cluster 2 (31)	Cluster 3 (2)	LSD	Sig
DTP	**9.38**	**9.84**	3.82	0.97	^∗∗∗^
HTW	**164.31**	129.18	60.93	25.31	^∗∗∗^
LT	**25.95**	17.96	13.51	2.80	^∗∗∗^
NRT	5.52	5.84	5.50	0.56	Ns
NT	14.08	13.23	6.64	5.17	Ns
NTS	39.44	39.43	30.47	16.76	Ns
PG	**70.57**	**73.30**	17.22	13.53	^∗∗∗^
PH	**99.73**	**92.89**	38.55	8.45	^∗∗∗^
TS	**1.88**	1.24	1.18	0.18	^∗∗∗^
WT	13.85	14.49	11.65	1.19	Ns

*Note:* Acronyms for traits are defined in Table 1; LSD: least significant difference at 5% level of probability. The bold values indicate the traits that have significant contributions at each cluster.

^∗∗∗^: *p* ≤ 0.001.

**Table 5:** Factor analysis and predicted selection gain based on the multitrait genotype-ideotype distance index (MGIDI).

Variables	FA1	FA2	FA3	FA4	Communality	Uniqueness	Goal	PSG (%)	Sense
PG	0.07	0.03	0.02	**0.96**	0.92	0.08	100	0.42	Increase
NT	**−0.83**	−0.11	−0.14	0.18	0.75	0.25	100	14.3	Increase
DTP	−0.043	−0.16	0.46	**0.56**	0.74	0.26	100	5.74	Increase
PH	−0.16	−0.26	0.04	**0.90**	0.90	0.10	100	0.96	Increase
NTS	**−0.70**	0.14	−0.04	−0.07	0.51	0.49	100	1.68	Increase
NRT	0.13	−0.08	**0.73**	−0.06	0.57	0.43	100	5.68	Increase
LT	0.02	**−0.93**	0.28	0.12	0.96	0.04	100	12.40	Increase
WT	0.01	−0.03	**0.86**	0.14	0.76	0.24	100	2.75	Increase
TS	0.04	**−0.97**	−0.12	0.07	0.96	0.04	100	8.85	Increase
HTW	−0.51	**−0.53**	0.39	0.22	0.75	0.25	100	22.80	Increase
Average					0.78	0.22			
Total increase								75.58	
Total decrease								—	

*Note:* Acronyms for traits are defined in Table 1; FA1–FA4: factor analysis. The bold values indicate the traits that have significant contributions at each FA.

Abbreviation: PSG = predicted selection gain.

**Table 6:** Factor analysis of the selected six tiger nut genotypes based on multitrait genotype-ideotype distance index (MGIDI).

Variables	FA1	FA2	FA3	FA4	Communality	Uniqueness
PG	0.36	0.37	**0.77**	0.26	0.92	0.08
NT	−0.12	0.01	0.00	−0.99	1.00	0.00
DTP	**0.97**	0.18	−0.05	0.06	0.97	0.03
PH	−0.20	**0.92**	0.26	−0.03	0.97	0.03
NTS	0.70	−0.25	0.21	0.16	0.62	0.38
NRT	0.25	0.03	−0.92	0.27	0.99	0.01
LT	−0.51	0.43	−0.32	0.66	0.99	0.01
WT	**0.90**	0.03	−0.34	0.27	1.00	0.00
TS	−0.75	0.31	−0.09	**0.56**	0.98	0.02
HTW	0.09	**0.98**	−0.05	0.15	1.00	0.00

*Note:* Acronyms for traits are defined in Table 1; FA1–FA4: factor analysis.

## Data Availability

The data that support the findings of this study are available from the corresponding authors upon reasonable request.

## Supporting Information

Supporting Table 1: list of tiger nut genotypes evaluated. Supporting Table 2: mean values of the evaluated tiger nut genotype for 10 agronomic and tuber yield traits.
