# Supplementary material for: Multitrait Selection for Higher Agronomic and Tuber Yield–Related Traits in Tiger Nut (Cyperus esculentus L.) Genotypes
Source: Scientifica (Cairo). 2025 Jun 23;2025:9458568. doi: 10.1155/sci5/9458568 (PMC12208757; doi:10.1155/sci5/9458568)
Supplement: Supporting Information — Additional supporting information can be found online in the Supporting Information section. [file 9458568.f1.docx]

**Supplementary Information**

Supplementary Table 1. List of tiger nut genotypes evaluated

| S/N | Accession | Colour | Place/ Area of collection | Region |
| --- | --- | --- | --- | --- |
| *1* | ADU- b | *Brown* | *Aduamoa* | *Eastern* |
| *2* | ADU- B | *Black* | *Aduamoa* | *Eastern* |
| *3* | ADU- Y | *Yellow* | *Aduamoa* | *Eastern* |
| *4* | APR- b | *Brown* | *Assin Praso* | *Central* |
| *5* | ASU- b | *Brown* | Asukese No.2 | *Eastern* |
| *6* | BAJ- b | *Brown* | Bawjiase | *Central* |
| *7* | BAJ- B | *Black* | Bawjiase | *Central* |
| *8* | BAJ- Y | *Yellow* | Bawjiase | *Central* |
| *9* | BAW- b | *Brown* | Bawku | *Upper East* |
| *10* | BEP- B | *Black* | Beposo | *Central* |
| *11* | BEP- Y | *Yellow* | Beposo | *Central* |
| *12* | BUO- b | *Brown* | Buoyam | *Bono East* |
| *13* | BUO- B | *Black* | Buoyam | *Bono East* |
| *14* | DED- b | *Brown* | Dedeso | *Eastern* |
| *15* | DEM- b | *Brown* | Demso | *Eastern* |
| *16* | ENK- b | *Brown* | Enkroful | *Central* |
| *17* | ENK- B | *Black* | Enkroful | *Central* |
| *18* | GYI- b | *Brown* | Gyilli | *Upper East* |
| *19* | KAL- b | *Brown* | Kaleo | *Upper East* |
| *20* | KAL- B | *Black* | Kaleo | *Upper East* |
| *21* | MBA- b | *Brown* | Mbaabaasa | *Central* |
| *22* | MBA- B | *Black* | Mbaabaasa | *Central* |
| *23* | NEB- b | *Brown* | New Ebu | *Central* |
| *24* | OBR- b | *Brown* | Obratwawu | *Central* |
| *25* | OBR- B | *Black* | Obratwawu | *Central* |
| *26* | OBR- Y | *Yellow* | Obratwawu | *Central* |
| *27* | OEB- b | *Brown* | Old Ebu | *Central* |
| *28* | OEB- B | *Black* | Old Ebu | *Central* |
| *29* | OFF- b | *Brown* | Offinso | *Eastern* |
| *30* | PUT- b | *Brown* | Putobio | *Central* |
| *31* | PUT- B | *Black* | Putobio | *Central* |
| *32* | SAN- b | *Brown* | Sankana | *Upper East* |
| *33* | TAK- b | *Brown* | Takpo | *Upper East* |
| *34* | TAK- B | *Black* | Takpo | *Upper East* |
| *35* | TWI- b | *Brown* | Twifo Praso | *Central* |
| *36* | TWI- Y | *Yellow* | Twifo Praso | *Central* |
| *37* | WIO- b | *Brown* | Wiomua | *Central* |
| *38* | WIO- B | *Black* | Wiomua | *Central* |
| *39* | YEN- b1 | *Brown* | Yendi | *Northern* |
| *40* | YEN- b2 | *Brown* | Yendi | *Northern* |
| *41* | YEN- b3 | *Brown* | Yendi | *Northern* |
| *42* | ZEB- Y | *Yellow* | Zebila | *Upper West* |

Supplementary Table 2. Mean values of the evaluated tiger nut genotypes for 10 agronomic and tuber yield traits

| Genotype | DTP | HTW | LT | NRT | NT | NTS | PG | PH | TS | WT |
| --- | --- | --- | --- | --- | --- | --- | --- | --- | --- | --- |
| ADU- b | 11.53 | 182.97 | 26.46 | 6 | 14.93 | 28.73 | 86.67 | 103.6 | 1.89 | 13.95 |
| ADU- B | 8.4 | 132 | 18.75 | 6 | 10.07 | 42.07 | 94.44 | 101.6 | 1.13 | 16.7 |
| ADU- Y | 9.6 | 113.44 | 22.46 | 6 | 8.13 | 12.93 | 91.11 | 95.13 | 1.72 | 13.12 |
| APR- b | 9.93 | 186.02 | 22.99 | 5 | 22.73 | 27.93 | 74.44 | 95.53 | 1.69 | 13.39 |
| ASU- b | 11.47 | 185.2 | 26.46 | 6.33 | 18.2 | 42.13 | 57.78 | 104.2 | 1.81 | 14.57 |
| BAJ- b | 10 | 114.08 | 18.3 | 5.33 | 12 | 34.07 | 41.11 | 76.6 | 1.14 | 16.06 |
| BAJ- B | 9.6 | 131.21 | 16.53 | 6 | 9.67 | 21.87 | 85.56 | 83.27 | 1.08 | 15.25 |
| BAJ- Y | 4.23 | 78.44 | 14.43 | 5 | 10.67 | 47.93 | 22.22 | 49.3 | 1.08 | 13.39 |
| BAW- b | 8.73 | 177.22 | 8.82 | 5 | 24.2 | 61.73 | 85.56 | 104.8 | 1.02 | 8.66 |
| BEP- B | 11.4 | 143.36 | 17.99 | 6 | 22.2 | 39.13 | 62.22 | 88.6 | 1.31 | 13.72 |
| BEP- Y | 10.47 | 86.07 | 15.45 | 5 | 21.73 | 20.6 | 47.78 | 82.6 | 1.16 | 13.43 |
| BUO- b | 8.47 | 126 | 30.81 | 6 | 13.6 | 43.07 | 88.89 | 113.33 | 2.01 | 15.35 |
| BUO- B | 9.2 | 175.41 | 19.86 | 6 | 16.6 | 45.93 | 74.44 | 100.07 | 1.15 | 17.22 |
| DED- b | 10.87 | 136.02 | 30.3 | 6 | 14.27 | 38.47 | 77.78 | 99.33 | 2.45 | 12.4 |
| DEM- b | 8.87 | 180.11 | 25.46 | 5.33 | 10.13 | 51.2 | 63.33 | 94.6 | 1.9 | 13.62 |
| ENK- b | 10.47 | 133.27 | 19.68 | 6 | 13.53 | 38.73 | 45.56 | 93.27 | 1.32 | 14.89 |
| ENK- B | 12.6 | 174.4 | 18.16 | 6 | 17.67 | 58.87 | 87.78 | 97.93 | 1.16 | 15.77 |
| GYI- b | 10.13 | 100.74 | 15.15 | 6 | 14.27 | 24.33 | 57.78 | 82.07 | 1.02 | 14.77 |
| KAL- b | 9.8 | 78 | 16.65 | 5 | 11.8 | 35.33 | 82.22 | 95.5 | 1.24 | 13.3 |
| KAL- B | 9.73 | 105.63 | 17.6 | 6 | 12 | 33.33 | 98.89 | 97.67 | 1.12 | 15.75 |
| MBA- b | 9.47 | 162.46 | 24.26 | 6 | 10.8 | 40.07 | 61.11 | 84.07 | 1.64 | 14.75 |
| MBA- B | 9.87 | 174.13 | 17.8 | 6 | 13.13 | 57.8 | 60 | 90.47 | 1.09 | 16.35 |
| NEB- b | 7.4 | 119.02 | 17.99 | 6 | 9.87 | 20 | 82.22 | 105.47 | 1.42 | 12.54 |
| OBR- b | 10.53 | 169.53 | 23.53 | 5 | 16.13 | 33.13 | 90 | 104 | 1.74 | 13.33 |
| OBR- B | 11.3 | 138.21 | 23.04 | 6 | 14.73 | 49.73 | 66.67 | 86.4 | 1.7 | 14.37 |
| OBR- Y | 9.93 | 118.1 | 18.24 | 5.33 | 11.73 | 33.33 | 95.56 | 110.33 | 1.14 | 15.94 |
| OEB- b | 3.4 | 43.42 | 12.58 | 6 | 2.6 | 13 | 12.22 | 27.8 | 1.27 | 9.91 |
| OEB- B | 10.33 | 163.04 | 19.37 | 6.33 | 11.53 | 46.87 | 48.89 | 77 | 1.18 | 16.25 |
| OFF- b | 8.93 | 201.65 | 28.69 | 6 | 11.6 | 33.6 | 76.67 | 112.8 | 2.08 | 14.41 |
| PUT- b | 12.27 | 159.07 | 23.39 | 6.67 | 14.73 | 41 | 63.33 | 83.4 | 1.45 | 16.01 |
| PUT- B | 9.87 | 171.24 | 17.29 | 6 | 11.47 | 38.6 | 85.56 | 104.07 | 1.07 | 16.16 |
| SAN- b | 8.77 | 113.14 | 19.49 | 6 | 8.67 | 30.4 | 76.67 | 101.67 | 1.32 | 14.81 |
| TAK- b | 10.53 | 135.15 | 19.66 | 6 | 12 | 28.73 | 81.11 | 96.07 | 1.26 | 15.48 |
| TAK- B | 10.07 | 119.41 | 18.6 | 6 | 12.53 | 47.67 | 71.11 | 92.6 | 1.18 | 15.8 |
| TWI- b | 8 | 167.06 | 25.69 | 5.33 | 11.73 | 54.27 | 41.11 | 92.67 | 1.72 | 14.58 |
| TWI- Y | 9.33 | 118.96 | 13.98 | 5.67 | 10.47 | 51.27 | 73.33 | 86.93 | 1.04 | 13.47 |
| WIO- b | 7.33 | 129.44 | 19.59 | 5 | 11.57 | 44.53 | 36.28 | 81.7 | 1.44 | 13.65 |
| WIO- B | 7.89 | 120.31 | 16.1 | 6 | 14.4 | 40.4 | 43.33 | 60.6 | 1.11 | 14.5 |
| YEN- b1 | 10.13 | 93.41 | 15.45 | 5 | 10.27 | 37.87 | 100 | 111.67 | 1.25 | 12.46 |
| YEN- b2 | 8.07 | 97.39 | 15.97 | 5.33 | 9.2 | 27.4 | 85.56 | 99.33 | 1.12 | 14.23 |
| YEN- b3 | 7.67 | 31.02 | 9.78 | 6 | 14.07 | 75.67 | 76.67 | 89.2 | 1.23 | 7.96 |
| ZEB- Y | 10.4 | 120.47 | 14.51 | 6 | 8.33 | 44.47 | 88.89 | 97.07 | 0.98 | 14.88 |
